# Clinical Significance and Prognostic Value of Hemostasis Parameters in 337 Patients with Acute Infective Endocarditis

**DOI:** 10.3390/jcm10225386

**Published:** 2021-11-18

**Authors:** Rosa Zampino, Domenico Iossa, Maria Paola Ursi, Lorenzo Bertolino, Arta Karruli, Rosa Molaro, Gennaro Esposito, Martina Vitrone, Fabiana D’Amico, Rosina Albisinni, Emanuele Durante-Mangoni, on behalf of the Monaldi Hospital CardiovascularInfection Group

**Affiliations:** 1 Department of Advanced Medical and Surgical Sciences, University of Campania “Luigi Vanvitelli”, 80138 Naples, Italy; rosa.zampino@unicampania.it (R.Z.); mariapaola.ursi@hotmail.com (M.P.U.); lorenzo.bertolino91@gmail.com (L.B.); gennaro.esposito6@studenti.unicampania.it (G.E.); martina.vitrone@yahoo.it (M.V.); 2 Unit of Infectious & Transplant Medicine, A.O.R.N. Ospedali dei Colli-Ospedale Monaldi, 80131 Naples, Italy; Domenico.iossa@unicampania.it (D.I.); fabianadamico@libero.it (F.D.); rosa.albisinni@libero.it (R.A.); 3 Department of Precision Medicine, University of Campania “Luigi Vanvitelli”, 80131 Naples, Italy; arta.karruli@unicampania.it (A.K.); rossellamolaro@libero.it (R.M.)

**Keywords:** prognosis, embolism, D-dimers, aPTT, biomarkers

## Abstract

(1) Background: The aim of this study was to assess the clinical significance and prognostic role of the main hemostasis parameters in infective endocarditis (IE): prothrombin time as international normalized ratio (PT-INR), activated partial thromboplastin time (aPTT), fibrinogen, D-dimers, platelet count, homocysteine. (2) Methods: We studied 337 patients with IE. Clinical, hemato-chemical and echocardiography parameters were analyzed. Coagulation parameters were measured on admission. (3) Results: D-dimers levels (*p* = 0.012) and a prolonged PT-INR (*p* = 0.013) were associated with higher in-hospital mortality, while prolonged aPTT (*p* = 0.021) was associated with increased 1-year mortality. *Staphylococcus aureus* (*S. aureus*) infection (*p* = 0.003), prosthetic valve endocarditis (PVE) (*p* = 0.001), surgical indication (*p* = 0.002) and higher D-dimer levels (*p* = 0.005) were independent predictors of in-hospital mortality. PVE (*p* = 0.001), a higher Charlson Comorbidity Index (*p* = 0.049), surgical indication (*p* = 0.001) and prolonged aPTT (*p* = 0.012) were independent predictors of 1-year mortality. Higher levels of D-dimers (*p* < 0.001) and a shorter aPTT (*p* < 0.001) were associated with embolic complications of IE. *S. aureus* etiology was bound to higher D-dimers levels (*p* < 0.001) and a shorter aPTT (*p* = 0.006). (4) Conclusions: Elevated D-dimers are associated with a higher risk for in-hospital mortality in IE patients. High D-dimers and a short aPTT are associated with a higher risk for embolic events in IE. A longer aPTT is associated with 1-year mortality.

## 1. Introduction

Infective endocarditis (IE) is a potentially fatal disease with a mortality rate of over 20%, largely unmodified over recent decades [1,2]. Mechanisms of IE pathogenesis are still incompletely understood but involve bacteria, host immune responses and the coagulation system [3,4,5,6].

Vegetations, the pathologic hallmark of IE, consist of a fibrin mesh wherein platelets and bacteria are embedded. Vegetations form as a consequence of a pathological thrombo-inflammatory reaction and underlie the progression of the disease and its complications, including heart valve destruction and septic embolic events [7,8]. The first step in vegetation formation encompasses an abnormal immune thrombotic reaction with subsequent bacterial entrapment [4,9]. Among major IE causative pathogens, *Staphylococcus aureus* directly promotes coagulation, generating thrombin-like activity by staphylocoagulase and von Willebrand factor (vWF)-binding protein, making up staphylothrombin [10]. Staphylothrombin mediates the conversion of fibrinogen to fibrin and initiates platelet (PLT) aggregation [11]. Intriguingly, the inhibition of staphylothrombin by dabigatran was safe and effective in reducing coagulation activation, time to blood culture clearance and metastatic foci of infection in *S. aureus* bacteremia, including IE [12].

These data strongly suggest it could be beneficial to monitor and modulate coagulation in IE. Among coagulation parameters, D-dimers have been previously identified as a marker able to provide important insights into the infection-related coagulation abnormalities [13,14,15]. Three studies have evaluated the prognostic role of D-dimers in IE. Turak and colleagues suggested that high D-dimers levels on admission could identify IE patients at increased risk for in-hospital mortality [16]. Lin et al. found D-dimers as a prognostic factor of in-hospital adverse events and six-month mortality [17], while Xu et al. found that higher plasma D-dimers levels were predictive of ischemic stroke in 173 patients with IE [18]. However, use of coagulation markers in the prediction of IE prognosis has not yet entered into clinical practice [19].

Therefore, the primary aim of this study was to assess whether routine hemostasis parameters (PLT count, prothrombin time as international normalized ratio (PT-INR), activated partial thromboplastin time (aPTT), fibrinogen and D-dimers) have prognostic value for in-hospital and 1-year mortality in patients with IE. The secondary objective was to analyze the possible association of hemostasis markers with IE clinical features, microbial etiology, comorbidities and complications.

## 2. Materials and Methods

### 2.1. Study Design

In this retrospective study, we included patients with a diagnosis of definite IE, admitted between 2007 and 2019 at the Unit of Infectious and Transplant Medicine, Monaldi Hospital, University of Campania “Luigi Vanvitelli”. IE diagnosis was made according to the current criteria (modified Duke criteria until 2014, and ESC criteria from 2015 on) [19,20]. In the study period, we observed 525 IE cases in our hospital, a regional referral center for IE. Out of these, we included in this study all patients (*n* = 337) admitted to our unit with a recent diagnosis of IE (definite or possible) without the need for emergency surgery. Complete data were not available for all enrolled patients, as some coagulation parameters were not recorded in our database in the first years of the accrual time; the number of patients for whom each parameter was available is specified in Table 1.

This study was approved by the Ethics Committee of the University of Campania “Luigi Vanvitelli” and AORN Ospedali dei Colli (prot. N. AOC/011110/2020). Informed consent was obtained from patients for the anonymous collection and use of their clinical data.

### 2.2. Patients

Data of patients were available as part of a standardized protocol of IE evaluation followed at our unit, which includes a baseline clinical assessment, particularly of clinical history, physical examination, chest X-ray, abdominal ultrasound scan and hemato-chemical tests (including full blood count, PT-INR, aPTT, fibrinogen, D-dimers, C-reactive protein (CRP), creatinine, urea, glycemia, alanine transferase (ALT)). According to the protocol, a trans-thoracic echocardiogram (TTE) was performed in all patients within 72 h of admission, followed by a transesophageal echocardiogram (TEE) where needed. Detailed information about IE characteristics (native or prosthetic valve, or cardiac implantable electronic device (CIED)), endocardial vegetations (number, size and position) and isolated causative pathogens was also collected. Embolic events, defined as acute complications causing overt clinical manifestations [21], and their characteristics (location, extension, complications) were also recorded.

The Charlson Comorbidity Index (CCI) was calculated for each patient, and the estimated glomerular filtration rate (eGFR) was computed by the Modification of Diet in Renal Disease (MDRD) formula. Anticoagulant treatment is also reported.

### 2.3. Laboratory Assays

Hemato-chemical parameters were obtained by routine methods used in our hospital’s central laboratory and included main hemostasis parameters (prothrombin time as international normalized ratio (PT-INR), activated partial thromboplastin time (aPTT), fibrinogen, D-dimers, platelet count, homocysteine), and biochemical markers, such as CRP and troponin. Hemato-chemical parameters used for this study were collected on hospital admission.

### 2.4. Statistical Analysis

Numerical data are presented as the median with the interquartile range, and categorical/nominal data as the number and percentage. The Mann–Whitney U test and the Kruskal–Wallis test were used to assess the statistical significance of differences between two or more subgroups of numerical variables. Fisher’s exact test was used to compare subgroups for nominal variables. Logistic regression analysis of independent predictors of hospital and 1-year mortality was performed by block entering in the model all variables significantly associated with each of these outcomes in univariate analysis. To assess the predictive performance of hemostasis parameters on IE outcome, we calculated the area under the receiver operating characteristic (ROC) curve, entering in-hospital mortality as the state variable.

The significance level was set at 5%, and all tests were 2-tailed. All analyses were performed using the statistical software for Windows SPSS 20 (SPSS, Inc., Chicago, IL, USA).

## 3. Results

### 3.1. Clinical Features and Coagulation Parameters of the Study Population

Clinical features and coagulation parameters of the 337 IE patients studied are shown in Table 1. The median age was 64 years, and 70.3% (n = 237) were males. The most frequent causative pathogens of IE were staphylococci and streptococci. The aortic valve and native heart valves were the most frequent sites affected by the infection. In this cohort, the in-hospital mortality rate was 9.5%, 1-year mortality was 22.7% and 70 patients (21%) were lost to long-term follow-up.

Data on anticoagulant treatment on admission were available for 307 patients, of whom 80 were treated with warfarin, 17 were heparin-treated patients and 210 were not on anticoagulant treatment (*p* = 0.103).

### 3.2. Hemostasis Parameters and IE Mortality

Coagulation parameters were evaluated in relation to in-hospital and 1-year mortality of IE patients (Table 2). In-hospital mortality was associated with higher D-dimers levels (*p* = 0.012) and prolonged PT-INR (*p* = 0.013), while 1-year mortality was associated with prolonged aPTT (*p* = 0.021).

In multivariate logistic regression analysis, *S. aureus* infection (*p* = 0.003), prosthetic valve endocarditis (PVE) (*p* = 0.001), indication for surgery (*p* = 0.002) and higher D-dimers levels (*p* = 0.005) were independently associated with in-hospital mortality (Table 3A). Moreover, PVE (*p* = 0.001), higher CCI (*p* = 0.049), surgical indication (*p* = 0.001) and prolonged aPTT (*p* = 0.012) were independent predictors of 1-year mortality (Table 3B).

Regarding anticoagulation treatment, no significant differences were observed regarding in-hospital mortality between patients treated with anticoagulants (14/97) and those not treated with anticoagulants (17/210; *p* = 0.103). There was also no significant difference in 1-year mortality between anticoagulant-treated (26/97) and untreated (43/210; *p* = 0.24) patients.

**Figure 1:**
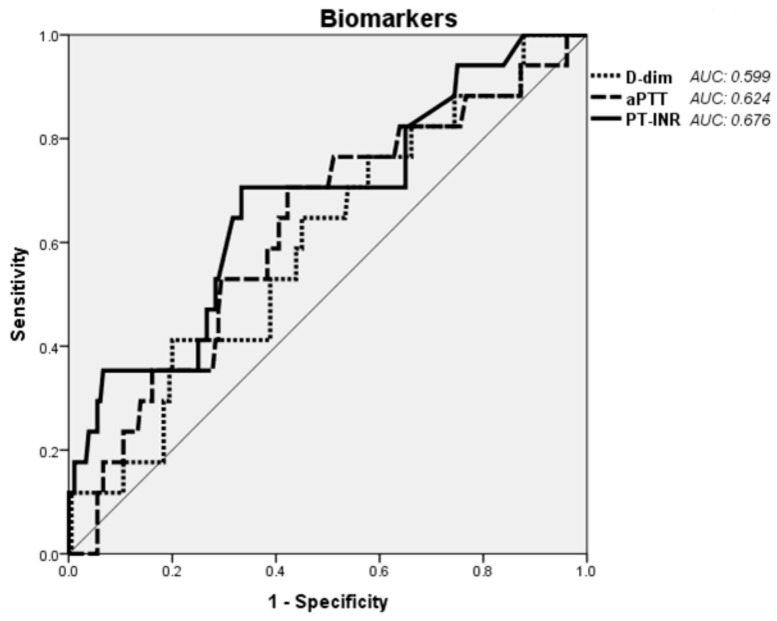
Receiver operating characteristic curve analysis of the predictive performance of three hemostasis parameters on in-hospital mortality of IE. Abbreviations: D-dim: D-dimers; aPTT: activated partial thromboplastin time; PT-INR: prothrombin time international normalized ratio.

The predictive performance of hemostasis parameters for in-hospital mortality as assessed by the area under the ROC curves appeared to be fair (Figure 1).

The area under the receiver operating characteristic curve for each parameter is shown on the graph. Predictive power for the designated outcome was low for all three hemostasis parameters assessed.

### 3.3. Hemostasis Parameters and IE Clinical Features

Coagulation parameters were analyzed in relation to major IE clinical features (Table 2). Older age was associated with higher D-dimers levels (*p* = 0.047). Embolic events occurred in 104 patients (30.9%) and were associated with higher levels of D-dimers (*p* < 0.001) and shorter aPTT (*p* < 0.001). Surgery was indicated in 246 of 337 patients (73%), was actually performed in 208 of them (84%) and showed an association with higher PT-INR levels (*p* = 0.013; Table 2).

Regarding the type of infection (native vs. prosthetic valve vs. CIED), PT-INR was predictably higher in patients with PVE (*p* < 0.001). However, aPTT was also significantly more prolonged in the latter patient subgroup (*p* = 0.008). The PLT count was lower in right-sided IE, whilst CIED-related IE patients had the highest homocysteine levels. Median vegetation size in this patient subset was 15 mm, and D-dimers were significantly higher in patients showing a vegetation size between 10 and 20 mm (*p* = 0.041; Supplementary Table S1).

**Figure 2:**
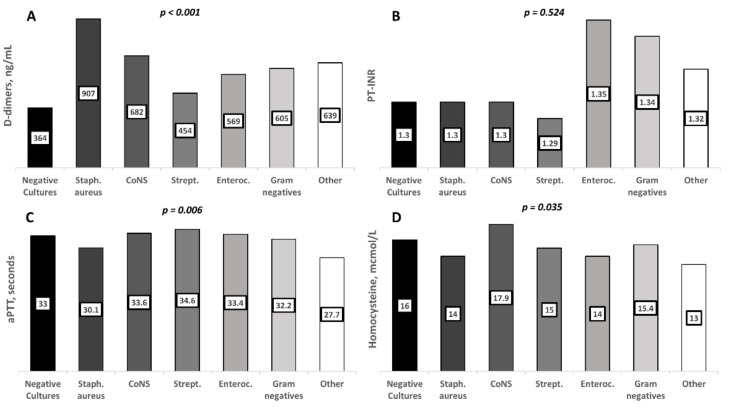
Hemostasis parameters according to the major subgroups of IE causative pathogens. Columns indicate median values for each subgroup: (**A**) D-dimers; (**B**) PT-INR; (**C**) aPTT; (**D**) homocysteine. Abbreviations: CoNS: coagulase-negative staphylococci; Strept: streptococci; Enteroc: *Enterococci*; PT-INR: prothrombin time international normalized ratio; aPTT: activated partial thromboplastin time.

Concerning IE etiology, *S. aureus* was associated with higher D-dimers (*p* < 0.001) and shorter aPTT levels (*p* = 0.006; Figure 2). No other associations were observed in relation to the etiology of IE.

Median values of the analyzed parameters in each study subgroup are shown within the relevant bars. Significant associations were observed for higher D-dimers levels and shorter aPTT in *S. aureus* IE and a higher homocysteine level in IE due to coagulase-negative staphylococci.

### 3.4. Hemostasis Parameters and Comorbidities

The most common comorbidities in the 337 IE patients studied were chronic heart failure and diabetes mellitus, followed by chronic liver disease and chronic kidney disease (Table 1).

Two hundred and fifteen (63.8%) patients presented at least one comorbidity. The median CCI was 5 (range 3–7). Table 4 shows coagulation parameters in relation to CCI. D-dimers and fibrinogen levels did not change according to CCI. In contrast, a J-shaped relationship was observed between CCI and the remaining hemostasis parameters. Patients with CCI ≤1 or ≥4 had a prolonged PT-INR (*p* = 0.006), and a trend for a longer aPTT (*p* = 0.057), a lower PLT count (*p* = 0.089) and a higher homocysteine level (*p* = 0.060) (Table 4).

## 4. Discussion

Coagulation and inflammation are closely linked, and a prothrombotic tendency is often observed in acute infectious diseases [9,22]. In the present study, we evaluated the association of hemostasis parameters with the severity and prognosis of IE. Being inexpensive routine tests, easy to obtain in every hospital, coagulation parameter assessments could improve the clinical management of IE.

Derived from one of the largest single-center cohorts of IE where hemostasis has been studied, our data suggest that coagulation parameters are strictly tied to the course and outcome of the disease. D-dimers were independently associated with in-hospital mortality, whereas aPTT was a predictor of 1-year mortality. In addition, higher levels of D-dimers and shorter aPTT, both signaling a pro-coagulant state, translated into a higher risk of embolic events. In contrast, the significance of PLT and fibrinogen levels remains unclear from our data.

Among coagulation parameters, D-dimers have received the most interest, and their use in clinical practice is well established. Our results corroborate previous data suggesting a predictive value of D-dimers for the short-term prognosis of IE and the prediction of embolism [16,17,23,24] and septic embolic stroke [18]. Consistently, Meini et al. recently found that very high levels of D-dimers signaled a worse in-hospital outcome in patients with acute, severe infections [13].

Interestingly, aPTT levels also showed predictive value for IE outcomes. Their association with 1-year mortality needs to be elaborated as, to the best of our knowledge, no prior similar data exist. Apparently, a shorter aPTT implies a prothrombotic state that associates with IE embolic complications and staphylococcal etiology. Whether this relates to factor VIII or vVF levels remains to be studied. In contrast, longer aPTT may be associated with other forms of coagulopathy that signal a reduced chance of survival. PT-INR and aPTT are modified by anticoagulant treatment in patients with prosthetic valves or atrial fibrillation, and it is well known that PVE and arrhythmias imply a worse IE outcome and a higher incidence of embolism [25,26,27]. Overall, it is interesting to note that common coagulation parameters may provide more clinically relevant information in IE than other parameters that are more complex to obtain, such as homocysteine [28] and prothrombotic genetic polymorphisms [29].

Etiology also appeared to have an impact on coagulation function in IE. *S. aureus* was significantly associated with higher D-dimers levels and shorter aPTT, confirming and further supporting previous data about the role of *S. aureus* in the coagulation imbalance in IE [10,11]. Further investigation of the role of staphylothrombin inhibition by direct anticoagulants in *S. aureus* IE [12] seems warranted.

Comorbidities were common in our cohort, and a CCI ≥ 4 was associated with specific changes in hemostasis parameters. Since previous studies highlighted the negative effect of comorbidities on IE outcome [30,31,32], we hypothesize that changes in coagulation function could represent one possible link between these two factors.

Our study has some limitations. First, it was a retrospective study. Second, some patients were referred to our unit from other facilities and were often treated before the diagnosis of IE, possibly altering the kinetics of coagulation parameters measured at hospital admission. Third, patients who underwent emergency surgery on hospital admission were excluded, thus leaving the more severe ones out of this study. Fourth, there was a sizeable amount of missing data, and a considerable number of patients were lost to the 1-year follow-up.

In conclusion, elevated D-dimers are associated with a higher risk for in-hospital mortality in IE patients. High D-dimers and a short aPTT are associated with a higher risk for embolic events in IE. A longer aPTT is associated with 1-year mortality. Hemostasis parameters appear to be inexpensive tools that are easy to obtain, which are able to improve the evaluation of IE prognosis.

## Figures and Tables

**Table 1:** Baseline characteristics of the study group.

Parameter	Missing Data	Result
Patient number	-	337
Age, years	-	64 (51–73)
Male gender	-	237 (70.3)
Chronic heart failure (prior to IE onset)	-	100 (29.7)
Charlson Comorbidity Index	139	5 (3–7)
Diabetes mellitus	-	62 (18.4)
Chronic hepatitis	-	61 (18.1)
Chronic kidney disease (Stages 3–5)	-	59 (17.5)
Platelet count, cells 10^3^/μL	-	203 (149–262)
Creatinine, mg/dL	-	1.0 (0.8–1.3)
Troponin I, ng/mL	59	0.05 (0.02–0.53)
D-dimers, ng/mL	32	605 (296–1101)
PT-INR	-	1.3 (1.18–1.3)
Fibrinogen, mg/dL	134	407 (322–532)
aPTT, seconds	5	32.5 (29.7–38.1)
Homocysteine, mcmol/L	82	15 (12–20)
C-reactive protein, mg/dL	2	5.2 (2.4–9.9)
Vegetation location - Aortic valve - Mitral valve - Tricuspid/pulmonary valve - Cardiac implantable electronic device - Multivalve involvement - Other	2	115 (34.3) 79 (23.6) 25 (7.5) 86 (25.7) 28 (8.4) 2 (0.6)
IE type: - Native valve - Prosthetic valve - Cardiac implantable electronic device - Other	2	145 (43.3) 93 (27.8) 86 (25.7) 11 (3.3)
IE causative pathogen: - *Streptococci* - Coagulase-negative *Staphylococci* - *Staphylococcus aureus* - *Enterococci* - Negative cultures - Gram negatives - Other pathogens	-	97 (28.8) 63 (18.7) 53 (15.7) 52 (15.4) 49 (14.5) 14 (4.2) 9 (2.7)
Vegetation size (max. dimension), mm (n. 257)	80	14 (9–20)
Embolic event	-	104 (30.9)
Cardiac surgery	-	208 (62.3)
In-hospital mortality	-	32 (9.5)
1-year mortality	70	76 (22.6)

Data are expressed as median and interquartile range (IQR) or number and percentages (%). Abbreviations: IE: infective endocarditis; PT-INR: prothrombin time international normalized ratio; aPTT: activated partial thromboplastin time.; n.: number of patients with available data.

**Table 2:** Coagulation parameters in relation to major clinical features and mortality of IE patients.

	D-Dimers	*p*-Value	Fibrinogen	*p*-Value	PT-INR	*p*-Value	aPTT	*p*-Value	PlateletCount	*p*-Value	Homocysteine	*p*-Value
Gender:		0.246		0.775		0.058		0.741		0.728		0.357
Male	566 (280–1086)		402 (313–536)		1.30 (1.15–1.50)		32.6 (30–38.1)		205 (150–262)		15 (12–20)	
Female	673 (345–1167)		433 (332–532)		1.40 (1.20–2.17)		32.5 (29.4–37.8)		200 (137–269)		14 (12–19)	
Age:		**0.047**		0.099		0.270		0.729		**0.001**		**0.003**
≤64	558 (257–1015)		417 (333–567)		1.30 (1.14–1.50)		32.7 (29.2–37.8)		215 (162–285)		14 (11–18.5)	
>64	633 (358–1217)		390 (300–501)		1.30 (1.19–1.82)		32.5 (29.6–38.4)		190 (131–239)		16 (13–22)	
Embolic event:		**<0.001**		0.134		0.473		**<0.001**		0.150		0.366
No	512 (237–995)		402 (306–515)		1.30 (1.17–1.80)		33.7 (30.6–38.9)		204 (146–258)		15 (12–20)	
Yes	765 (532–1592)		446 (338–585)		1.30 (1.18–1.50)		30.6 (28.4–34.8)		203 (153–286)		14.3 (11.7–18.1)	
Surgery indication:		0.309		0.595		**<0.001**		0.266		0.917		0.840
No	525 (310–1027)		433 (364–504)		1.41 (1.21–2.35)		33.7 (29.6–39)		206 (150–261)		15.2 (12–19)	
Yes	617 (296–1167)		399 (308–555)		1.29 (1.12–1.50)		32.1 (29.6–37.3)		203(148–265)		14.5 (12–20)	
Surgery performed:		0.164		0.252		**<0.001**		0.230		0.782		0.523
No	525 (286–965)		424 (331–555)		1.40 (1.20–2.20)		33.7 (29.1–39.1)		204 (151–261)		15.1 (12–19)	
Yes	627 (312–1196)		397 (302–528)		1.29 (1.12–1.50)		32.1 (29.9–37.3)		203 (147–266)		14.2(11.4–20.2)	
Hospitalization outcome:		**0.012**		0.933		**0.013**		0.090		0.398		0.886
Alive	582 (274–1041)		407 (324–535)		1.30 (1.15–1.70)		32.3 (29.6–37.8)		204 (152–262)		15 (12–20)	
Dead	869 (498–1927)		419 (299–527)		1.50 (1.22–2.6)		36.6 (31.5–43)		201 (103–279)		15 (12–22)	
1-year outcome:		0.390		0.821		0.078		**0.021**		0.275		0.059
Alive	567 (315–1028)		424 (331–56)		1.30 (1.15–1.60)		32.3 (30.1–37.6)		209 (154–275)		14.2 (11.4–19)	
Dead	667 (290–1326)		438 (322–546)		1.30 (1.20–2.28)		36.6 (31.5–44)		201 (143–266)		16 (12–22)	

Data are median (IQR); *p*-value was generated by Mann–Whitney U test; 246 surgery indication–208 surgery performed. Abbreviations: aPTT: activated partial thromboplastin time; PT-INR: prothrombin time international normalized ratio. Statistically significant results are marked in bold.

**Table 3:** Parameters associated with in-hospital mortality (panel A) and 1-year mortality (panel B).

A						
Parameter	In-Hospital Mortality		Univariate Analysis		Multivariate Logistic Regression Analysis	
	**Survived** **(*n* = 305**)	**Deceased** **(*n* = 32)**	**Odds Ratio (95% C.I.)**	***p*-Value ^**	**Odds Ratio (95% C.I.)**	***p*-Value**
Age	64 (50–73)	68 (55–75)		0.113		
Gender:						
Male	213 (69.84)	24 (75)	0.77 (0.33–1.78)	0.685		
Female	92 (30.16)	8 (25)				
IE etiology:				**0.004**	**1.57 (1.16–2.13)**	**0.003**
*S. aureus*	47 (15.4)	6 (18.75)				
Coagulase-negative *Staphylococci*	55 (18.03)	8 (25)				
*Streptococci*	95 (31.1)	2 (6.25)				
*Enterococci*	48 (15.7)	4 (12.5)				
Gram negatives	10 (3.2)	4 (12.5)				
Other pathogens	6 (1.9)	3 (9.3)				
Negative cultures	44 (14.4)	5 (15.7)				
Infection type:	(n = 218)	(n = 20)	4.10 (1.51–11.10)	**0.004**	**10.49 (2.79–39.48)**	**0.001**
Native	139 (63.7)	6 (30)				
Prosthetic	79 (36.3)	14 (70)				
Charlson Comorbidity Index	(n = 180) 5 (3–7)	(n = 18) 6 (4–7)		0.253		
Embolic event *:			1.19 (0.55–2.57)	0.689		
Yes	93 (30.5)	11 (34.4)				
No	212 (69.5)	21 (65.6)				
Intracardiac abscess			2.34 (0.98–5.58)	0.059		
Yes	38 (12.4)	8 (25)				
No	267 (87.6)	24 (75)				
Surgery indication:	(n = 303)	(n = 32)	3.83 (1.13–12.90)	**0.020**	**14.07 (2.68–73.80)**	**0.002**
Yes	217 (71.6)	29 (90.6)				
No	86 (28.4)	3 (9.4)				
D-dimers, ng/mL	582 (274–1041)	869 (498–1927)		**0.012**	**1.00 (1.00–1.00)**	**0.005**
Fibrinogen, mg/dL	407 (324–535)	419 (299–527)		0.933		
PT-INR	1.30 (1.15–1.70)	1.50 (1.22–2.6)		**0.013**	1.61 (0.94–2.74)	0.077
aPTT, seconds	32.3 (29.6–37.8)	36.6 (31.5–43)		0.090		
Platelet count, cells 10^3^/μL	204 (152–262)	201 (103–279)		0.398		
Homocysteine, mcmol/L	15 (12–20)	15 (12–22)		0.886		
**B**						
**Parameter**	**1-Year Mortality**		**Univariate Analysis**		**Multivariate Logistic** **Regression Analysis**	
	**Survived** **(*n* = 191)**	**Deceased** **(*n* = 76)**	**Odds Ratio (95% C.I.)**	***p*-Value ^**	**Odds Ratio (95% C.I.)**	***p*-Value**
Age	62 (49–73)	65 (55–75)		0.058		
Gender:			0.81 (0.45–1.46)	0.558		
Male	130 (68.1)	55 (72.3)				
Female	61 (31.9)	21 (27.7)				
IE etiology:				**0.017**	**1.34 (0.92–1.96)**	**0.123**
*S. aureus*	25 (13.1)	12 (15.8)				
Coagulase-negative *Staphylococci*	34 (17.8)	18 (23.7)				
*Streptococci*	69 (36.1)	12 (15.8)				
*Enterococci*	34 (17.8)	11 (14.5)				
Gram negatives	6 (3.1)	5 (6.6)				
Other pathogens	3 (1.6)	3 (3.9)				
Negative cultures	20 (10.5)	15 (19.7)				
Infection type:	(n = 141)	(n = 51)	2.18 (1.13–4.17)	**0.020**	**12.90 (3.02–55.09)**	**0.001**
Native	93 (66)	24 (47)				
Prosthetic	48 (34)	27 (53)				
Charlson Comorbidity Index	(n = 110) 5 (2–7)	(n = 36) 6 (4.2–7)		**0.009**	**1.32 (1.00–1.74)**	**0.049**
Embolic event *:			0.76 (0.42–1.37)	0.461		
Yes	61 (32)	20 (26.3)				
No	130 (68)	56 (73.7)				
Intracardiac abscess			1.37 (0.66–1.84)	0.439		
Yes	25 (15.5)	13 (17.1)				
No	136 (84.5)	63 (82.9)				
Surgery indication:			3.83 (1.13–12.90)	**0.005**	**2.59 (4.515–235.17)**	**0.001**
Yes	133 (70)	66 (86.8)				
No	57 (30)	10 (13.2)				
D-dimers, ng/mL	567 (315–1028)	667 (290–1326)		0.390		
Fibrinogen, mg/dL	424 (331–567)	438 (322–546)		0.821		
PT-INR	1.30 (1.15–1.60)	1.30 (1.20–2.28)		0.078		
aPTT, seconds	32.3 (30.1–37.6)	36.6 (31.5–44)		**0.021**	**1.09 (1.02–1.17)**	**0.012**
Platelet count, cells 10 ^3^/μL	209 (154–275)	201 (143–266)		0.275		
Homocysteine, μmol/L	14.2 (11.4–19)	16 (12–22)		0.059		

Data are presented as median with interquartile range (IQR) or as number and percentage (%). ^ The *p*-value was generated by Fisher’s exact test or Pearson chi-square test for categorical variables, and Mann–Whitney for numerical variables; * includes stroke. Abbreviations: aPTT: activated partial thromboplastin time; IE: infective endocarditis; PT-INR: prothrombin time international normalized ratio. Statistically significant values are marked in bold.

**Table 4:** Coagulation parameters according to the Charlson Comorbidity Index in IE patients.

CI	0–1 (*n* = 22)	2–3 (*n* = 34)	≥4 (*n* = 142)	*p*-Value ^
D-dimers, ng/mL	452 (215–866)	649 (337–1037)	614 (259–1222)	0.439
Fibrinogen, mg/dL	439 (383–605)	423 (355–580)	409 (306–537)	0.323
PT-INR	1.35 (1.25–1.58)	1.20 (1.09–1.37)	1.33 (1.20–2.14)	**0.006**
aPTT, seconds	33.2 (29.3–38.2)	30.9 (29–34)	33 (30.1–38.4)	0.057
Platelet count, cells10^3^/μL	209 (149–302.7)	229 (183–280)	201 (139–259)	0.089
Homocysteine, mcmol/L	14 (11–16.2)	12 (11–18)	15 (12–21)	0.060

Data are median (IQR). ^^^ Kruskal–Wallis test. Abbreviations: aPTT: activated partial thromboplastin time; PT-INR: prothrombin time international normalized ratio; CCI: Charlson Comorbidity Index; IE: infective endocarditis. Statistically significant results are marked in bold.

## Data Availability

The dataset used for this study is available on request to the corresponding author.

## Supplementary Materials

The following are available online at https://www.mdpi.com/article/10.3390/jcm10225386/s1, Supplementary Table S1: Coagulation parameters according to, site of infection, type of infection and vegetation size in IE patients.

## Appendix A

Members of the Monaldi Hospital Cardiovascular Infection Study Group:

Emanuele Durante-Mangoni MD, PhD; Domenico Iossa PhD; Lorenzo Bertolino MD; Maria Paola Ursi MD;, Fabiana D’Amico BSc; Arta Karruli MD; Mohammad Ramadan MD; Roberto Andini MD; Rosa Zampino MD (Internal Medicine); Mariano Bernardo MSc; Giuseppe Ruocco MSc (Microbiology); Giovanni Dialetto MD; Franco Enrico Covino MD; Sabrina Manduca MD (Echocardiography); Alessandro Della Corte MD, PhD; Luca S. De Santo MD, PhD; Antonio Carozza MD, PhD; Marisa De Feo MD, PhD (Cardiac Surgery); Stefano De Vivo MD (Electrophysiology); Maria Luisa De Rimini MD (Nuclear Medicine); Nicola Galdieri MD (Intensive Care Unit).
