# Supplementary material for: Clinical Significance and Prognostic Value of Hemostasis Parameters in 337 Patients with Acute Infective Endocarditis
Source: J Clin Med. 2021 Nov 18;10(22):5386. doi: 10.3390/jcm10225386 (PMC8624946; doi:10.3390/jcm10225386)
Supplement: Supplementary file 1 [file jcm-10-05386-s001.zip › jcm-1431678-supplementary.pdf]

Journal of
*Clinical Medicine*

MDPI

# Supplementary Materials

**Supplementary Table S1.** Coagulation parameters according to the location of the vegetation, type of infection and vegetation size in IE patients.

| | **Aortic valve (*n* = 115)** | **Mitral valve (*n* = 79)** | **Tricuspid/Pulmonary valve (*n* = 25)** | **CIED (*n* = 86)** | **Multivalve (*n* = 28)** | **Others (*n* = 2)** | **^*p*-value** |
|---|---|---|---|---|---|---|---|
| D-dimer, ng/mL | 528 (266–1012) | 672 (286–1290) | 750 (355–2372) | 612 (267–1128) | 602 (454–982) | 787 (787) | 0.531 |
| Fibrinogen, mg/dL | 417 (331–527) | 436 (319–556) | 452 (312–615) | 386 (282–492) | 401 (292–631) | 402 (402) | 0.728 |
| PT-INR | 1.3 (1.2–1.7) | 1.3 (1.17–1.9) | 1.44 (1.17–1.9) | 1.27 (1.1–1.67) | 1.3 (1.18–1.43) | 1.51 (1.51) | 0.514 |
| aPTT, seconds | 32.9 (29.18–38.18) | 32.4 (30.4–36.8) | 32.2 (29.3–37) | 32.7 (29.7–37.68) | 35.3 (30–38.8) | 27.7 (27.7) | 0.701 |
| Platelet, cells*10³/µL | 217 (159–286) | 209 (140–275) | 170 (142–219) | 175 (140–225) | 218 (137–341) | 206 (192) | **0.049** |
| Homocysteine, µmol/L | 14 (12–20) | 14 (11–17) | 14 (9.4–17.3) | 16 (12.4–21) | 13 (11–19) | 13 (13) | 0.273 |

| | **Native valve (*n* = 145)** | **Prosthetic valve (*n* = 93)** | **CIED (*n* = 86)** | **Others (*n* = 11)** | **^*p*-value** |
|---|---|---|---|---|---|
| D-dimer, ng/mL | 627 (324–1159) | 716 (340–1286) | 612 (267–1128) | 392.5 (237.5–1008) | 0.804 |
| Fibrinogen, mg/dL | 416 (324–571) | 435 (331–522) | 386 (282–492) | 402 (317–502) | 0.407 |
| PT-INR | 1.26 (1.12–1.40) | 1.74 (1.22–2.6) | 1.27 (1.10–1.67) | 1.4 (1.2–1.56) | **<0.001** |
| aPTT, seconds | 31.4 (29.4–35.3) | 35.7 (30.4–42.65) | 32.7 (29.7–37.68) | 34.1 (29.2–37.75) | **0.008** |
| Platelet, cells*10³/µL | 206.5 (153–283) | 206 (165–294) | 184 (140–245) | 203.5 (178.5–535.5) | 0.096 |
| Homocysteine, µmol/L | 14 (11–18.9) | 15 (12–20.5) | 16 (12.4–21) | 12 (8–14) | **0.020** |

| **Vegetation size** | **0–10 mm (*n* = 92)** | **10.1–20 mm (*n* = 114)** | **20.1–30 mm (*n* = 40)** | **>31 mm (*n* = 11)** | **^*p*-value** |
|---|---|---|---|---|---|
| D-dimer, ng/mL | 528 (258–1013) | 792 (409–1545) | 689 (370–1339) | 581 (377–875) | **0.041** |
| Fibrinogen, mg/dL | 392 (285–504) | 402 (343–583) | 388 (297–507) | 330 (285–430) | 0.417 |
| PT-INR | 1.26 (1.16–1.48) | 1.27 (1.14–1.53) | 1.3 (1.2–1.4) | 1.3 (1–1.4) | 0.903 |
| aPTT, seconds | 32.3 (29.2–41.4) | 32.9 (29.1–36.8) | 31 (29.6–34.9) | 31 (29.08–34.4) | 0.526 |
| Platelet, cells*10³/µL | 203 (141–273) | 201 (149–262) | 197 (150–259) | 153 (120–252) | 0.639 |

Values are median (IQR); ^Kruskal-Wallis test; Abbreviations: aPTT: activated partial thromboplastin time; PT-INR: Prothrombin time International Normalized Ratio; CIED: Cardiovascular Implantable Electronic Device. Statistically significant results are marked in bold
